# Cardiac Phase Space Tomography: A novel method of assessing coronary artery disease utilizing machine learning

**DOI:** 10.1371/journal.pone.0198603

**Published:** 2018-08-08

**Authors:** Thomas D. Stuckey, Roger S. Gammon, Robi Goswami, Jeremiah P. Depta, John A. Steuter, Frederick J. Meine, Michael C. Roberts, Narendra Singh, Shyam Ramchandani, Tim Burton, Paul Grouchy, Ali Khosousi, Ian Shadforth, William E. Sanders

**Affiliations:** 1 Cone Health Heart and Vascular Center, Greensboro, North Carolina, United States of America; 2 Austin Heart, Austin, Texas, United States of America; 3 Piedmont Heart Institute, Atlanta, Georgia, United States of America; 4 Rochester General Hospital, Rochester, New York, United States of America; 5 Bryan Heart, Lincoln, Nebraska, United States of America; 6 New Hanover Regional Medical Center, Wilmington, North Carolina, United States of America; 7 Lexington Cardiology, West Columbia, South Carolina, United States of America; 8 Atlanta Heart Specialists, Cumming, Georgia, United States of America; 9 Analytics 4 Life, Toronto, Ontario, Canada; 10 A4L (US), Morrisville, North Carolina, United States of America; Worcester Polytechnic Institute, UNITED STATES

## Abstract

**Background:**

Artificial intelligence (AI) techniques are increasingly applied to cardiovascular (CV) medicine in arenas ranging from genomics to cardiac imaging analysis. Cardiac Phase Space Tomography Analysis (cPSTA), employing machine-learned linear models from an elastic net method optimized by a genetic algorithm, analyzes thoracic phase signals to identify unique mathematical and tomographic features associated with the presence of flow-limiting coronary artery disease (CAD). This novel approach does not require radiation, contrast media, exercise, or pharmacological stress. The objective of this trial was to determine the diagnostic performance of cPSTA in assessing CAD in patients presenting with chest pain who had been referred by their physician for coronary angiography.

**Methods:**

This prospective, multicenter, non-significant risk study was designed to: 1) develop machine-learned algorithms to assess the presence of CAD (defined as one or more ≥ 70% stenosis, or fractional flow reserve ≤ 0.80) and 2) test the accuracy of these algorithms prospectively in a naïve verification cohort. This report is an analysis of phase signals acquired from 606 subjects at rest just prior to angiography. From the collective phase signal data, features were extracted and paired with the known angiographic results. A development set, consisting of signals from 512 subjects, was used for machine learning to determine an algorithm that correlated with significant CAD. Verification testing of the algorithm was performed utilizing previously untested phase signals from 94 subjects.

**Results:**

The machine-learned algorithm had a sensitivity of 92% (95% CI: 74%-100%) and specificity of 62% (95% CI: 51%-74%) on blind testing in the verification cohort. The negative predictive value (NPV) was 96% (95% CI: 85%-100%).

**Conclusions:**

These initial multicenter results suggest that resting cPSTA may have comparable diagnostic utility to functional tests currently used to assess CAD without requiring cardiac stress (exercise or pharmacological) or exposure of the patient to radioactivity.

## Introduction

The broad application of artificial intelligence (AI) techniques to all facets of medicine is rapidly changing practice economics as well as the available clinical tools for treatment and diagnosis [1–4]. In cardiovascular medicine, machine learning (ML) has been applied in arenas ranging from genomics to cardiac imaging analysis [1–2]. Recent refinements in the methods of machine learning, including deep learning/neural networks and cognitive computing, now permit the expansion of AI analysis beyond the traditional realm of “big data” outcomes to that of novel diagnostic tests [2, 5]. ML provides a pathway to improve the accuracy and reliability of diagnostic modalities, and has potential to significantly contribute, on multiple levels, to achieving the goal of precision medicine in the cardiovascular area.

Cardiac Phase Space Tomography Analysis (cPSTA) is a novel method to assess the presence of significant CAD in major coronary arteries, defined as ≥ 70% stenosis by angiography or ≤ 0.80 fraction flow reserve (FFR). A hand-held device collects a patient’s resting phase signals without the use of ionizing radiation, contrast agents, exercise, or pharmacologic stressors. The acquired 10 million data points are transferred to the cloud and evaluated by an analytic engine (CAD AE) employing machine-learned algorithms/predictors. The results are subsequently displayed as a phase space tomography model, which is accessible via a web portal.

We now report the development of machine-learned algorithms for assessing CAD and the performance of the final predictors in a blindly tested cohort compared to angiography results. These findings are an analysis of the initial algorithm development and validation stage (Stage I) of the ongoing Coronary Artery Disease Learning & Algorithm Development (CADLAD) trial [6].

## Methods

### Study design

Stage I of the CADLAD trial entailed two distinct steps. First, a prospective, non-randomized development step was conducted to generate machine-learned algorithms for assessment of the presence of significant coronary artery disease (CAD) using paired phase signals with clinical outcomes data. Second, a blinded, paired comparison was performed to verify the performance of the machine-learned algorithm from step 1 with regard to the assessment of significant CAD as determined by coronary angiography, which represents the “gold standard” for evaluation of CAD. A patient was considered CAD positive for development purposes if at least one of the two following criteria were fulfilled: 1) one lesion had a stenosis of ≥ 70% or 2) if at least one lesion has a reduced fractional flow reserve (FFR) of ≤ 0.80. This is consistent with the accepted definition of clinically significant diameter narrowing of a coronary artery [7–8]. Based on the American College of Cardiology (ACC) guidelines [7]. FFR evaluation was the final determinate of CAD in vessels undergoing flow wire assessment.

In order to accurately machine learn, the results of a known standard must be paired with a signal to develop a solution that correctly reflects actual physiologic status. In this trial, all subjects (N = 606) had been referred for coronary angiography, and all subjects underwent the planned catheterization procedure with dye injections of both right and left coronary arteries. The CADLAD trial was designed to utilize the results of coronary angiograms as the “standard” and paired these catheterization findings with phase signals for the purpose of machine learning. Thereby, the machine-learning platform developed solutions to assess the presence of CAD. This method required enrollment of only subjects already referred by their physician for coronary angiography.

An objective of the protocol was that the results should reflect a broad cross section of clinical practice, and this goal prompted selection of hospitals and clinics that mirrored the diverse array of facilities providing care to patients with heart disease. The aim was attainment of machine-learned solutions that could be generalized. The physicians participating in the protocol followed angiogram interpretation consistent with the ACC guidelines for determining CAD including the use of flow wire when recommended [7]. Employing this well-established categorization of lesion significance replicates the current general cardiology practice with reference to interpretation.

Written informed consent was obtained from all participants in this trial. The individuals in this manuscript have given written informed consent (as outlined in PLOS consent form) to publish these case details. The non-significant risk protocol for the CADLAD trial was approved by a central institutional review board (Western IRB) and performed at 12 enrolling centers in the United States. All study sites were community-based hospitals with expertise in interventional cardiology.

### Study participants

All participants had suspected but not known CAD and had been referred by their physicians for nonurgent angiography. Additional inclusion criteria included age > 21 years old and the ability to understand protocol requirements as well as provide informed consent. Exclusion criteria were prior documented myocardial infarction (MI) or previous percutaneous coronary interventions (PCI), prior coronary artery bypass grafting (CABG), indication for invasive coronary angiography other than to assess for obstructive CAD (e.g. arrhythmia, cardiomyopathy, valvular abnormality), previous heart valve replacement, previous sustained or paroxysmal atrial or ventricular arrhythmia, infiltrative myocardial disease (amyloid, sarcoid, right ventricular dysplasia), presence of cardiac implantable electronic device, including implantable cardioverter defibrillator, pacemaker, implantable loop recorders and other monitors, implantable neuro-stimulators, congenital heart disease, breast implants, pregnancy, breast feeding, currently taking any Type IA, IC or III antiarrhythmic, any history of amiodarone therapy, clinically significant chest deformity (e.g., pectus excavatum or pectus carinatum), and neuromuscular disease if the condition resulted in tremor or muscle fasciculations.

### System and device description

**Fig 1:**
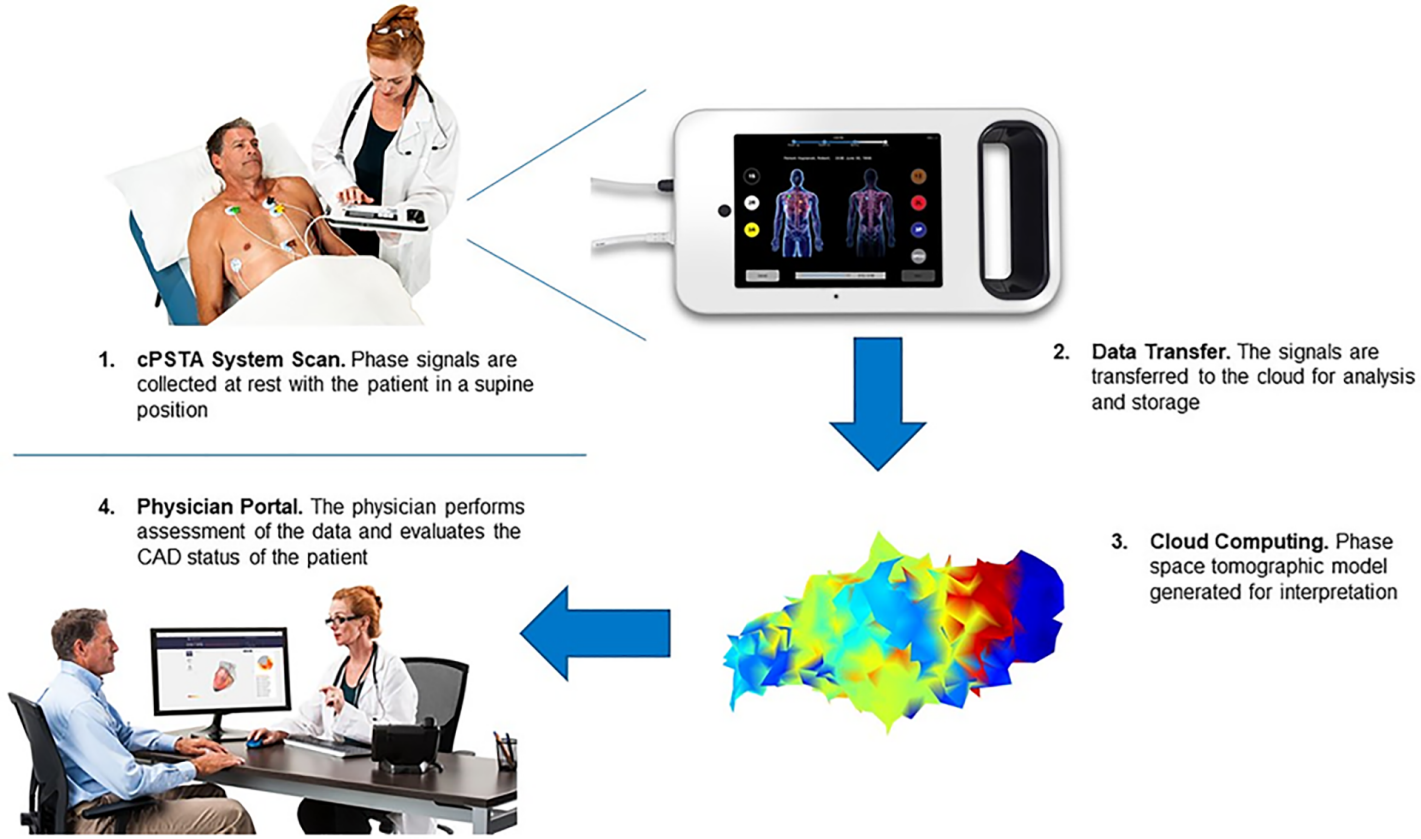
Utilization of the Cardiac Phase Space Tomography Analysis (cPSTA) System. Phase signal data are collected and transferred to cloud. The generated models and analysis are available for physician assessment. cPSTA System = Cardiac Phase Space Tomography Analysis System, CAD = coronary artery disease. Reprinted from presentation materials of A4L under a license, with permission from A4L and W20, original production 2016.

The cPSTA System is a medical device system that uses tomography to analyze phase signals and assess the presence of significant coronary artery disease in the major coronary arteries (Fig 1). The first component is the Phase Signal Acquisition (PSAQ) System which includes: 1) the phase signal recorder (PSR), a hand-held instrument that acquires and transmits resting phase signals along with ancillary patient-specific information (gender, age, etc.); and 2) a cloud-based phase signal data repository (PSDR) that accepts, stores, and allows retrieval of the signals as well as ancillary patient-specific information. The second component is a coronary artery disease analytical engine (CAD AE) that processes and evaluates the phase signals to assess the presence and significance of coronary artery disease using the machine-learned algorithms. The final component is Cloud Services (CS), including the health care provider (HCP) Web Portal that the clinician utilizes to interpret images, review results, and generate a report. The report can be saved as a record for inclusion in the patient’s medical record.

### Study procedures

**Fig 2:**
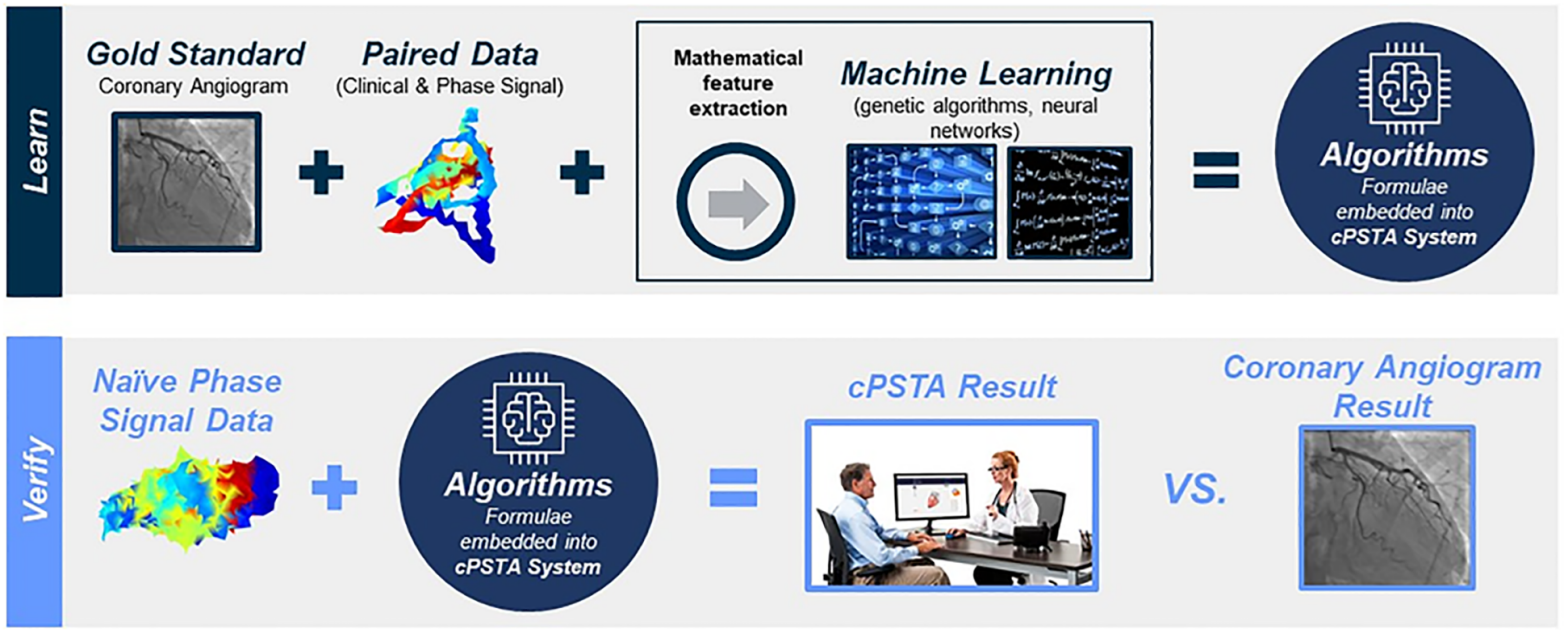
Development and verification of machine-learned predictor. The learning phase pairs “gold standard” results with phase signals for machine learning to develop algorithms. The verification phase tests the performance of the final algorithms on naïve signal data. cPSTA System = Cardiac Phase Space Tomography Analysis System. Reprinted from presentation materials of A4L under a license, with permission from A4L and W20, original production 2016.

After providing informed consent, participants underwent phase signal acquisition within seven days prior to angiography. Signals were captured utilizing the hand-held (PSR) device via seven sensors positioned on the chest and back. Phase signal data was collected for approximately 3 minutes and the data package was then transmitted wirelessly to the cloud based PSDR. An analytic engine, consisting of software based on the machine-learned algorithms, analyzed the acquired data and generated tomography images. The results can be made available through a secure web portal (Fig 2); however, in this trial, the outcomes from cPSTA were not provided to the physician caring for the participant, and no clinical decision-making was based on the cPSTA findings. All phase signals were procured by trained staff at each enrolling center. Angiography was performed by local physicians who made all clinical decisions regarding appropriate medical therapy. If the interventional cardiologist observed a lesion of questionable significance, use of a fractional flow wire was at his/her discretion. The participant’s involvement in the study was complete following acquisition of the phase signals and performance of the angiography. There was no required follow-up.

### Statistical analysis

Statistical analysis of the machine learned algorithm performance was done by direct calculation of the sensitivity, specificity, negative predictive value (NPV), and positive predictive value (PPV) using standard formulas. Bootstrap confidence intervals were used to assess the significance of these scores. Each subject was categorized into true negative (TN), true positive (TP), false negative (FN) and false positive (FP) by using a fixed, experimentally-determined threshold on the continuous output of the predictor, with an output below this threshold indicating a prediction of CAD negative; otherwise, the predictor is indicating CAD positive.

The 95% confidence intervals on these statistics were computed with the use of bootci, a MatLab R2016b (MathWorks; Natick, MA) function that returns the intervals employing bootstrap evaluation [9]. The receiver-operator characteristic curve (ROC) and its corresponding area under the curve (AUC) were computed with the use of the MatLab R2016b function perfcurve [10].

### Machine learning and algorithm development

The ML method employed was elastic net, which is a linear regression method with L1 and L2 regularization penalties [11]. The development set of signals (N = 512) was divided into separate training (N = 339) and validation (N = 173) sets. Elastic net was chosen because the combination of L1 and L2 penalties helps the approach overcome issues surrounding high-dimensional data with small training sets. In addition, it has been shown that linear regression techniques only need two independent observations per variable to avoid overfitting [12]. The elastic net received as input a set of features that represent tomographic and dynamical properties of the signal, along with the subject’s age, gender and heart rate. The features used in the machine-learned algorithms were extracted from each signal, meaning the signal is a sample and the features are components of the sample. A total of 405 features were used, with correlation-based feature reduction being applied before the linear model was learned (see below). When a high number of features are used for development of the model, dimensionality problems may arise with machine learning. High regularization methods can be used effectively to avoid overfitting and ameliorate the difficulties related to this issue [13]. Elastic net employed in our study automatically selects and regularizes the features to overcome the dimensionality issues. Elastic net has been shown to effectively train predictive models in high dimensional datasets with small sample sizes [14].

Additionally, the quality of subject’s signal was evaluated with a noise score (“noise volume”) designed to evaluate the impact of noise in the signal through changes in three-dimensional phase space by environmental factors such as complex powerline harmonics, medical imaging devices, and other electrical equipment in the proximity of the acquisition.

The elastic net algorithm learns a weight (*w*_*j*_) for each extracted feature (*f*_*j*_) with the final prediction for a given participant being calculated as

$$\sum_{j}w_{j}f_{j}+b$$

where *b* is the bias term. The L1 and L2 regularization methods penalized large weight values during learning, which helps prevent overfitting on the training set. Elastic net optimizes the above summation using the following loss function, incorporating both the fit of the output from the model as well as the regularization terms.

$$L(\alpha ,\lambda ,W)=∥y-XW{∥}^{2}+\lambda (0.5(1-\alpha ){∥W∥}_{2}^{2}+\alpha ∥W{∥}_{1})$$

$$∥W{∥}_{1}=\sum_{j=1}^{p}\left|w_{j}\right|$$

$${∥W∥}_{2}^{2}=\sum_{j=1}^{p}w_{j}^{2}$$

The parameters α and λ control the L1 and L2 penalties. Training was performed on a modified (continuous) Gensini score as the target [15], which applied severity and location scores for each lesion in the coronary tree and summed over the lesions to produce the score. This model only took into account the subject’s worst-case lesion (that with the maximal product of severity and lesion scores) and applied a logarithm such that it was more tractable for a linear model. A dampening factor of 0.25 was applied to the worst-case Gensini score when collaterals were present in order to increase the value of that modified Gensini score in predicting the subject’s true (binary) CAD label.

As with all machine learning algorithms, elastic net has several hyper-parameters, which modify the optimization process (e.g., α and λ). The implementation employed supported the upweighting of data points in the training set based on a threshold on the modified Gensini score, allowing for the adjustment of the importance of various subject categories during learning. An additional supported parameter is the reduction of the feature set by removing features that are correlated over a specific threshold. For the model reported here, a correlation threshold of 0.907 was used, reducing the feature set from 405 features to 315.

To automatically tune these various hyper-parameters, composed of weight, Gensini threshold for weighting, correlation reduction threshold, and two elastic net-specific hyper-parameters α and λ, a genetic algorithm (GA) was utilized [16,17]. At each generation of the GA, the performance of every genome (set of parameter values) in the current iteration was evaluated by inputting its values into elastic net, running elastic net, and measuring the final model’s performance on both the training and validation sets. The model’s continuous output of the modified Gensini score was treated as a predictor of the subjects’ binary CAD status. The model’s performance on these datasets was measured using the area under the receiver operator characteristic curve, so that all possible thresholds on the continuous output were considered. Since CAD is a continuous disease, with a large variety of lesion locations and sizes within the disease-positive and disease-negative cohorts, the modified Gensini score allowed the linear model to predict the disease along the natural continuum, and binning was only considered when assessing the fitness of the hyperparameters. Additionally, the fitness function allowed higher quality data, as measured by noise volume, to receive a higher weighting in the performance assessment. The fitness function is summarized in equation form below

$$Fitness=-1\times \sum_{i=1}^{5}{\left(1-AUC(T_{i})\right)}^{2}+1.5{\left(1-AUC(V_{i})\right)}^{2}$$

where T and V denote training and validation sets respectively and *i* denotes the noise subset as identified by thresholds on the noise volume. Five overlapping noise subsets are used as corresponding to four noise thresholds, where all data is contained in the largest noise set, but data above the first threshold was excluded from the second noise subset, and so on. Defining the fitness function to optimize the AUC on 10 different overlapping noise subsets from the development set has the advantage of avoiding overfitting any single set as well as optimizing the performance on all the sets.

Genomes whose parameters enabled elastic net to find good models relative to other genomes in the population were selected for reproduction to create new genomes. During reproduction, each parameter in a genome underwent “mutation” with a probability of 0.33, with mutations either perturbing the parameter by a random value or replacing it with a new random value. Half of all new genomes also underwent single-point crossover, where a random position on the genome was selected, with the parameters before this point coming from one high-performing genome from the previous generation, and the second set of parameters coming from a different, high-performing genome.

These new genomes become the population for the next generation, and the whole process repeated until no further improvements in fitness scores were observed for 10 consecutive generations of the GA. The resultant linear model that adequately assessed significant CAD was embedded in the analytic software and verified against the naïve cohort.

## Results

### Study population

Enrollment began in May 2016 and was completed in June 2017. There were 606 participants in the study, with 159 (31%) having protocol defined obstructive coronary lesions. The algorithm development cohort (training [N = 339] and validation [N = 173] sets) consisted of signals from 512 participants with an additional 94 participants’ signals serving as a naïve verification cohort (not utilized for machine learning purposes). The verification cohort was used for the blind testing of the machine-learned predictor. Demographics for the studied population are shown in Table 1. There was no significant difference between the development and verification cohorts except with regard to age of the subjects (p = 0.04). The entire population, both development and verification cohorts, included nearly 40% women. However, in this population presenting with chest pain and referred for angiography, only one third of participants had significant obstructive lesions as defined by the protocol at coronary angiography.

**Table 1:** Demographics of population.

Characteristics	Development (n = 512)	Verification (n = 94)	p-value
Mean Age—Years (Range)	61.5 ± 10.7	59.0 ± 9.8	0.04
Male (%)	60.2%	69.1%	0.11
Female (%)	39.8%	30.9%	0.11
Mean BMI (Range)	31.3 ± 7.0	32.5 ± 7.6	0.14
Diabetes Mellitus (%)	31.4%	35.1%	0.47
Hypertension (%)	72.9%	75.5%	0.70
Hypercholesterolemia/Hyperlipidemia (%)	71.3%	70.2%	0.90
Angiographic Results = CAD Negative (%)	69.1%	73.4%	0.46
Angiographic Results = CAD Positive (%)	30.9%	26.6%	0.46

### Performance of the machine-learned predictor

The final output of ML, the fixed algorithms incorporating features of the signal, were embedded within the CAD AE. Testing of the analytic engine of the cPSTA System blindly in the naïve verification cohort showed the final predictors had a sensitivity of 92% (95% CI: 74%-100%) and specificity of 62% (95% CI: 51%-74%) for the assessment of obstructive coronary artery disease [18]. There was an automatic selection of a clinically-relevant threshold from the ROC curve. The negative predictive value (NPV) was 96% (95% CI: 85%-100%) and the PPV = 46% (95% CI: 33%-62%).

### Safety of acquiring phase signals

The protocol anticipated the possibility of skin irritation or allergic reaction to the sensors used for collection of the phase signals. In 606 participants, no adverse events were reported.

## Discussion

Chest pain is ubiquitous, with studies showing a lifetime prevalence of 20–40% in the general population [19]. In those patients with chest pain that have been determined to have a pretest intermediate or high of coronary artery disease (CAD) by careful physical exam and history, functional (stress) diagnostic testing or computed tomographic angiography (CTA) is recommended [7]. Positive results from any of these tests presumably define the highest risk population for which coronary angiography may be required to determine if intervention is needed. In the United States, millions of stress tests are performed each year to evaluate patients presenting with complaints of stable chest pain [20–21]. Although simple treadmill testing with ECG evaluation may provide evidence of ischemia, cardiac stress testing with imaging comprises over 85% of the evaluations acquired, and the majority of tests are nuclear myocardial perfusion imaging (MPI) studies with single photon emission computed tomography (SPECT) [21]. This test typically entails exercising on a treadmill, injection of a radionuclide tracer, and two approximately 30-minute gamma camera scans, which occur 4 hours apart. Over seven million SPECT MPI studies are performed annually in the United States [21] at an average cost of $1000 [22]. Yet, in a recent study examining over 10,000 pretest intermediate risk patients, only 10–12% undergoing functional testing or CTA had positive results, and when those with post-test high probability of disease patients subsequently underwent angiography, less than half were shown to have significant obstructive CAD [23]. In large registry trials of patients without known heart disease who underwent angiography, less than 42% showed obstructive CAD [24]. The currently available screening imaging tests for CAD typically require physical or pharmacologic stress, commonly involve radiation exposure, and uniformly incur substantial cost. Functional testing and CTA are utilized in a gatekeeping fashion designed to risk stratify patients with chest pain and elucidate those individuals that would benefit from angiography [24–25]. Little has changed with regard to the accuracy of these technologies in the last decade, and better tools for screening CAD are needed.

In a community-based population presenting with chest pain and deemed by their physician to warrant angiography, we examined the utility of a novel form of tomography focusing on machine-learned predictors for the assessment of obstructive coronary arteries. The principle findings of this evaluation using data from Stage I of the CADLAD trial are 1) features extracted from phase signals can be employed in ML to develop final mathematical predictors that assesses the presence of significant CAD with an ROC-AUC = 0.80 (95% CI: 0.70, 0.88); 2) performance of the cPSTA is comparable to the most commonly employed functional test of MPI (Table 2); and 3) acquisition of phase signals is extremely safe and requires minimal patient time.

**Table 2:** Detecting flow-limiting CAD. Machine-Learned Predictor (cPSTA) Compared to Exercise SPECT [7] and Exercise ECG [7, 26].

Test	Sensitivity Range	Specificity Range
Rest cPSTA (N = 94)*	92% (95% CI = 74% to 100%)	62% (95% CI = 51% to 74%)
Exercise SPECT	82–88%	70–88%
Exercise ECG	54–75%	64–75%

* Negative Predictive Value for cPSTA was 96% (95% CI = 85% to 100%).

Artificial intelligence is rapidly evolving and its dramatic impact on physicians practice and the tools they utilize is beginning to take shape. In cardiovascular medicine during the last 10 years, ML techniques have been applied with success to disease diagnosis and prediction [1]. We report the development of machine-learned algorithms designed to assess the most commonly encountered cardiovascular disorder, coronary artery disease. The cPSTA, in this cohort, had a 92% sensitivity with a negative predictive value of 96%. The system was explicitly optimized (threshold chosen using the AUC-ROC curve) to maximize safety and, therefore sensitivity. The specificity of 62% remains comparable to other functional tests. Signals are collected with the patient at rest without radiation or stress, physical or otherwise.

Both MPI and CTA subject the patient to substantial radiation exposure ranging 8–12 mSv and 12–25 mSV for CTA and MPI, respectively [27]. Functional testing requires stress, physical or pharmacologic, which carries some risks including that of allergic reaction. Performance of CTA necessitates the use of contrast media, with potential deleterious renal effects. SPECT MPI has been performed for decades and remains the predominate imaging procedure utilized prior to cardiac catheterization. Practice patterns for evaluating chest pain that employ imaging studies have changed minimally over the years due to a lack of alternative technologies with equivalent or higher accuracy and enhanced safety.

ML is now an essential component for solving complex problems in most sciences and these same methods provide immense possibilities in medicine [28]. ML techniques can accommodate various “configurations of data, assign context weighting, and calculate the predictive power of every combinations of variables available” to evaluate the diagnostic elements [29]. We chose elastic net to learn linear models, as the L1 and L2 penalties help prevent overfitting, while linear regression requires only two independent observations per variable [12]. The main drawback with this approach is that it cannot learn potentially important nonlinear relationships between variables. As precision cardiovascular medicine evolves, machine-learned methods will increasingly provide diagnostic tools that permit better physician assessment of the individual patient’s condition, thereby augmenting safety and illuminating best care pathways. Machine learned solutions are being rapidly applied in cardiovascular medicine. Recently, lesion-specific ischemia, as assessed by invasive fractional flow reserve, was predicted by an integrated machine learning ischemia risk score based on quantitative plaque measures from CTA [30]. Wearable technologies that record cardiac function with embedded machine learned algorithms have demonstrated the capability to distinguished compensated from decompensated heart failure [31]. In the near future, new diagnostic tools based on machine learning will become widely available. Our analysis of Cardiac Phase Space Tomography suggests that machine-learned algorithms may offer a valuable new method for the assessment of patients with coronary artery disease.

## Study limitations

The development of machine learned algorithms and their subsequent incorporation into diagnostic tools presents multiple challenges. First, in this pilot trial the models were driven by a relatively small sample size. When machine learning with reduced sample sizes, overfitting must be avoided, and we utilized methods to overcome this potential source of error. Second, the algorithm was trained and evaluated in a specific population, patients with pretest intermediate to high risk of coronary artery disease. In order to generalize the framework, a larger and more heterogeneous data set would be required. Finally, the effects of environmental noise on the unfiltered signals and thus, the resultant predictors require further investigation in order to attain optimal models.

## Conclusions

The cPSTA System is a novel noninvasive tomographic imaging method for the detection of clinically significant CAD, which is based on machine learning. cPSTA exhibits comparable diagnostic performance to existing functional and anatomical modalities without the requirement of cardiac stress (exercise or pharmacological) and without exposure of the patient to radioactivity. This technology may provide a new and efficient technique for assessing the presence of obstructive coronary lesions in patients presenting with chest pain suspected to be of cardiac etiology.

## Supporting information

S1 File PONE-D-17-43044 Data.xlsx. (XLSX)
